# *De novo* transcriptome assembly of *Curvularia* sp. VKM F-3040, a promising steroid-modifying ascomycete

**DOI:** 10.1128/MRA.00663-23

**Published:** 2023-10-11

**Authors:** Vyacheslav V. Kollerov, Sergey V. Tarlachkov, Marina V. Donova

**Affiliations:** 1 G.K. Skryabin Institute of Biochemistry and Physiology of Microorganisms, Federal Research Center “Pushchino Center for Biological Research” of Russian Academy of Sciences Prospekt Nauki, Pushchino, Moscow region, Russia; University of Guelph, Guelph, Ontario, Canada

**Keywords:** *Curvularia* sp., 7-hydroxylation, steroid, transcriptome

## Abstract

This research presents *de novo* transcriptome shotgun assembly for *Curvularia* sp. VKM F-3040, which is a putative fungal strain able to modify androstane steroids with production of 7-hydroxy and 17-hydroxylated derivatives—key intermediates in the synthesis of pharmaceutical ingredients. The data are of importance for creating novel microbial biocatalysts.

## ANNOUNCEMENT

During our previous research on the diversity of steroid-modifying filamentous fungi, the ascomycete strain *Curvularia* sp. VKM F-3040 (syn. *Drechslera* sp. Ph F-34) was selected as the most promising biocatalyst of steroid substrate modifications capable of catalyzing 7α/β-hydroxylation and 17-keto-reduction (17β-HSD) (1), thus producing valuable 7-hydroxylated and 17 β-reduced steroid metabolites highly demanded by medicine to treat different illnesses (2 – 4). It was shown that the *Curvularia* sp. 7-hydroxylase is inducible, with the maximum inducing effect revealed for dehydroepiandrosterone (DHEA) (5). To identify the genes that may be involved in steroid structural modification by *Curvularia* sp., the *de novo* transcriptome shotgun sequencing and assembly have been performed.

The soil-originated strain *Curvularia* sp. VKM F-3040 was obtained from the All-Russian Collection of Microorganisms at the Institute of Biochemistry and Physiology of Microorganisms, Russian Academy of Sciences (VKM IBPM RAS). The mycelium of first-generation stage was grown in medium A (50 mL) containing (g/L) potato starch—45, glucose—5.0, and peptone—20 (рН 6.5) aerobically on a rotary shaker (220 rpm) at 28°C for 48 h in Erlenmeyer flasks (750 mL). Medium B containing (g/L) sucrose—40, peptone—10, K_2_HPO_4_—2, KH_2_PO_4_—16, MgSO_4_—0.5, FeSO_4_—0.05 (pH 6.5) was inoculated with aliquot of the first-generation mycelium (10% [vol/vol]) and incubated under conditions described above. After 18 h of incubation, the *Curvularia* sp. mycelium of second-generation stage was treated with 0.03% (wt/vol) DHEA in 2% (vol/vol) ethanol, whereas the control mycelium was treated by equal volume of ethanol only. Each variant was performed in two biological replicates. After 6 h of induction, the mycelium was collected by centrifugation of fermentation broth at 4,000 rpm for 40 min. The total RNAs were isolated from treated and untreated mycelium by using RNeasy Mini Kit reagent (Qiagen, USA) and purified using RNeasy Pure mRNA Bead Kit (Qiagen, USA), according to the manufacturer’s instructions to obtain mRNAs. Library was prepared by NEBNext Ultra II RNA Library Prep Kit (NEB, USA) and sequenced on an Illumina NextSeq 500 (Illumina, San Diego, CA, USA) to obtain single reads with a length of 76 and 83 bp. The quality of the reads was checked by FastQC v0.11.8 (6). Adapter sequences and low-quality regions in raw reads were removed using Trimmomatic v0.39 (7) with the following options: ILLUMINACLIP:TruSeq3-SE.fa:2:30:10; LEADING:5; SLIDINGWINDOW:4:15; and MINLEN:25. The remaining clean reads were used to assemble the transcriptome *de novo* applying Trinity v2.15.1 (8) with option --min_kmer_cov, 4. Pairwise similarity between gene sequences was determined using TaxonDC 1.3.1 (9) with default parameters.

Preliminary identification based on ITS1, 5.8S rRNA gene, ITS2, and partial 28S rRNA gene sequences revealed that the strain VKM F-3040 belongs to the *Curvularia* genus and is closest to *Curvularia spicifera*. A total of 149,438,693 single reads (12.0 Gbp) were obtained from the RNA sequencing. After trimming, 147,071,242 clean reads (11.5 Gbp) were *de novo* assembled into 17,133 contigs with a total length of 32,544,226 bp. The N50 of the transcriptome assembly is 2,981 bp, and the average contig length is 1,900 bp.

The obtained data open up the prospects for the identification of the key genes involved in steroid transformation in *Curvularia* sp. VKM F-3040, further creating novel microbial catalysts.

## Data Availability

The transcriptome shotgun assembly and the ITS1-5.8S-ITS2-28S region have been deposited in DDBJ/ENA/GenBank under the accession numbers GKMF00000000 and OR085838, respectively. The raw reads have been deposited to the NCBI Sequence Read Archive under the accession nos. SRR24745747, SRR24745746, SRR24745745, and SRR24745744.
